# Elevated prostate volume index and prostatic chronic inflammation reduce the number of positive cores at first prostate biopsy set: results in 945 consecutive patients

**DOI:** 10.1590/S1677-5538.IBJU.2019.0146

**Published:** 2020-10-30

**Authors:** Antonio B. Porcaro, Alessandro Tafuri, Marco Sebben, Giovanni Novella, Tania Processali, Marco Pirozzi, Nelia Amigoni, Riccardo Rizzetto, Aliasger Shakir, Matteo Brunelli, Maria Angela Cerruto, Filippo Migliorini, Salvatore Siracusano, Walter Artibani

**Affiliations:** 1 Department of Urology University of Verona Azienda Ospedaliera Universitaria Integrata Verona Verona Italy Department of Urology, University of Verona, Azienda Ospedaliera Universitaria Integrata Verona, Verona, Italy;; 2 Institute of Urology, and Catherine and Joseph Aresty Keck School of Medicine University of Southern California Los AngelesCA USA USC Institute of Urology, and Catherine and Joseph Aresty, Department of Urology, Keck School of Medicine, University of Southern California (USC), Los Angeles, CA, USA;; 3 Department of Pathology University of Verona Azienda Ospedaliera Universitaria Integrata Verona Verona Italy Department of Pathology, University of Verona, Azienda Ospedaliera Universitaria Integrata Verona, Verona, Italy

**Keywords:** Prostatic Neoplasms, Prostate, Prostate-Specific Antigen

## Abstract

**Objective:**

To assess the association between prostate volume index (PVI), and prostatic chronic inflammation (PCI) as predictors of prostate cancer (PCA). PVI is the ratio between the central transition zone volume (CTZV) and the peripheral zone volume (PZV).

**Materials and methods:**

Parameters evaluated included age, prostate specific antigen (PSA), total prostate volume (TPV), PSA density (PSAD), digital rectal exam (DRE), PVI, PCI and number of positive cores (NPC). All patients underwent baseline 14-core, trans-perineal random biopsies. Associations of parameters with the NPC were investigated by univariate and multivariate linear regression analysis.

**Results:**

Between September 2010 to September 2017, 945 patients were evaluated. PCA was detected in 477 cases (50.7%), PCI in 205 cases (21.7%). PCA patients, compared to negative cases, were older (68.3 vs. 64.4 years) with smaller TPV (36 vs. 48.3mL) and CTZV (19.2 vs. 25.4), higher PSAD (0.24 vs. 0.15ng/mL/mL), further PVI values were lower (0.9 vs. 1.18) and biopsy cores less frequently involved by PCI (9.4% vs. 34.2%).High PVI and the presence of PCI were independent negative predictors of NPC in model I considering PSA and TVP (PVI, regression coefficient, RC -0,6; p=0.002) and PCI (RC -1,4; p <0.0001); and in model II considering PSAD (PVI:RC -0,7; p <0,0001; and PCI: RC -1,5; p <0.0001).

**Conclusions:**

High PVI and the presence of PCI lowered the mean rate of NPC and is associated with less aggressive tumor biology expressed by low tumor burden. PVI can give prognostic information before planning baseline random biopsies. Confirmatory studies are required.

## INTRODUCTION

At present, prostate cancer (PCA) is a worldwide major health problem and is closely related to the male aging process ( 1 ). In daily practice, suspicion of PCA is a hard task for the urologist who is challenged to exclude or confirm the diagnosis by planning baseline random biopsies including cores taken from the apex to the base of the gland. Efforts have been applied in order to avoid unnecessary biopsies that represent the major drawback of this practice. Systematic baseline prostate biopsies give specific information of the microenvironment of the gland. When PCA is detected, positive cores are evaluated for site, zone, number, percentage of cancer involvement and tumor grade. As a result, clinical and pathological features allow tumor staging and classification of patients into classes with consequences on management because of their prognostic potential ( 1 ). On the other hand, histology of negative cores shows typical features including prostatic chronic inflammation (PCI), high grade intraepithelial neoplasia, glandular atrophy or hyperplasia ( 1 ).

Benign prostatic enlargement (BPE), which is histologically supported by a pattern of benign prostatic hyperplasia (BPH), PCI and PCA are diseases associated with aging. PSA is a useful marker for assessing total prostate volume (TPV), prostatic growth rate and PCA risk. Also, imaging evaluation of the prostate by measuring the volume is important when treating BPH by 5α-reductase inhibitors. So far DRE, PSA and prostate size are important parameters for assessing prostate diseases ( 1 , 2 ). However, the detection of abnormal findings will lead to the suspicion of PCA. A normal digital rectal exam (DRE) with prostate specific antigen (PSA) between 2 to 10ng/dL might suggest further investigations such as new biomarkers and imaging modalities in order to avoid unnecessary biopsies. Although promising, novel biomarkers do not show enough evidence to recommend their use in clinical practice, moreover, multiparametric resonance imaging (mp-MRI) should not be performed on baseline biopsies ( 1 , 2 ). In order to avoid unnecessary baseline biopsies, it is pivotal to assess clinical factors that associate with positive or negative cancer outcomes. Although PSA density (PSAD) has shown a positive association with the risk of PCA, especially in patients with PSA levels of 4-10ng/mL, it has limited predictive power because it closely depends on distributions of prostate volumes ( 1 , 3 ).

PCI has been classified into four categories by the National Institutes of Health ( 4 ). The last category, which is coded type IV, is detected after biopsy in patients who have no history of genitourinary tract pain complaints but present with increased levels of prostate-specific antigen (PSA) and/or abnormal digital rectal exam (DRE). Although the association between PCI and PCA is controversial, the majority of studies have shown that PCI reduces the risk of PCA ( 5 ).

In patients undergoing baseline prostate biopsies, our working group has demonstrated that PCI is inversely associated with the risk of PCA ( 6 - 12 ). Moreover, our group has also investigated the associations of prostate volume index (PVI), defined as the ratio of the volume of the central transition zone (CTZV) to the volume of the peripheral zone (PZV) of the prostate, with the risk of PCA and the outcomes have shown an inverse association ( 7 , 13 , 14 ).

The aim of this study was to evaluate both PVI and PCI as predictors of the number of positive cores in patients undergoing baseline biopsies.

## MATERIALS AND METHODS

The study had Institutional Board Review approval. All patients signed informed consent for using the data. Data of 1.910 patients were retrospectively evaluated during a period running from September 2010 to September 2017. The study evaluated patients elected to baseline random biopsies with PSA levels less than 30μg/L. Indications to perform biopsies were increased PSA levels, abnormal DRE or abnormal imaging of the prostate. Baseline biopsies were systemically taken in different zones of the gland according to the standard pattern including 14 cores. Analysis of adjunctive targeted cores were excluded in order to avoid skewing phenomena. Indications to perform prostate biopsies included increased PSA levels, abnormal DRE, increased PSA with abnormal DRE, and abnormal imaging findings.

Each patient was evaluated for age (years), body mass index (BMI, kg/m^2^), PSA (ng/L), DRE findings that were coded as normal or abnormal. Total volume of the prostate (TPV) and central transition zone volume (CTZV) were directly measured before biopsy by transrectal ultrasound (TRUS). In both cases, volume was measured by the formula for an ellipsoid [diameter1 x diemeter2 x diameter3 x 0.52] and transformed into volume (mL). The volume of the peripheral zone of the prostate (PZV) was measured by subtracting CTZV from TPV and PVI was calculated as the ratio of CTZV on PZV. PSAD was calculated as ratio of total PSA on TPV.

Each core was evaluated by our dedicated pathologist who systematically assessed the following features: (i) length (mm); (ii) ISUP tumour grade group; (iii) number of positive cores (from zero to 14); (iv) percentage of cancer involving each core; (v) prostatic Intraepithelial neoplasia (PIN); (vi) PCI; (vii) glandular atrophy; (viii) atypical small acinar cell proliferation. Features considered in this analysis were ISUP tumour grade group, number of cores involved by cancer and PCI was defined as type IV according to the definition of National Institutes of Health ( 4 ).

The aim and design of the study was to investigate, at baseline biopsies, the association of PVI and PCI, among other factors, with the prostate cancer extension assessed as tumour volume which was evaluated by considering the number of positive cores that ranged from zero (cores without cancer) to 14 (all cores involved by cancer). The number of cores sampled was not increased as total prostate volume increased.

## STATISTICAL METHODS

Summary statistics of population and subpopulations with or without the PCA were computed. Continuous variables were evaluated as means with relative standard deviations. Categorical factors were evaluated as frequencies with relative rates. Because of the non-normal distribution, continuous factors were transformed into natural logs in order to assess differences between groups and to compute linear regression analysis.

Differences of factors between groups were assessed by Student’s t test for continuous variables and by Chi squared test or Fisher’s exact test as appropriate for categorical factors. The association of factors with tumour extension was assessed by univariate and multivariate linear regression models considering the several factors as predictors of the NPC. Because of the high correlation between PSA, TPV and PSAD, two multivariate models were considered. Bivariate clinical models including PVI were computed. The software used to run the analysis was IBM-SPSS version 20. All tests were two-sided, with a significance level of p <0.05.

## RESULTS

**Figure 1:**
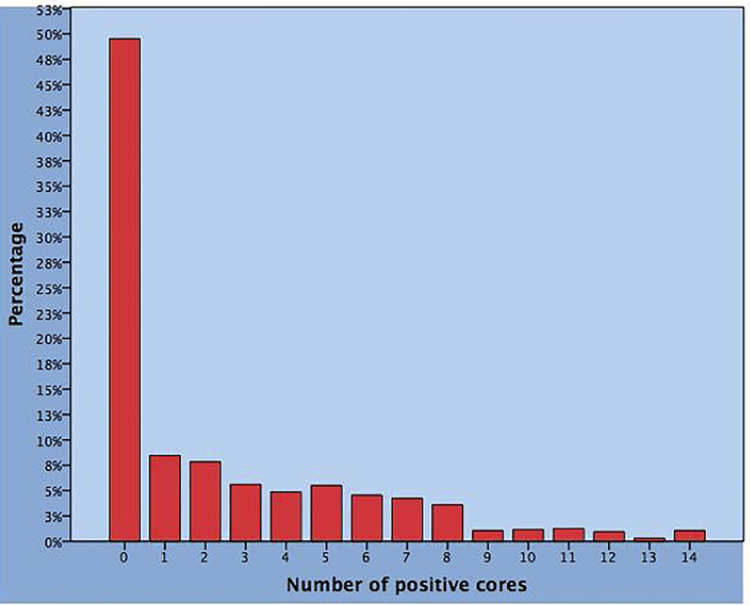
ercentages of negative and positive cores in 945 patients who underwent standard baseline trans-perineal biopsies because of suspected prostate cancer.

We evaluated 945 patients who met the inclusion criteria of the study. Statistics of the different parameters is reported in Table-1 . Percentages of negative and positive cores are depicted in Figure-1 . Overall, PCA was detected in 477 cases (50.7%) and the mean number of positive cores (NPC) was 4.7. The distribution of factors was significantly different (p <0.0001) between subgroups with or without PCA except for BMI (p=0.536). PCA patients, when compared to negative cases, were older (68.3 vs. 64.4 years) with higher PSA levels (7.8 vs. 6.6ng/mL), lesser prostate enlargements (lower measurements of TPV: 36 vs. 48.3mL, CTZV: 19.2 vs. 25.4, PZV: 19.2 vs. 22.), higher PSAD (0.24 vs. 0.15ng/mL/mL) and abnormal DRE more frequently detected (41.7 vs. 23.3); moreover, PVI values were lower (0.9 vs. 1.18) and cores less frequently involved by PCI (9.4% vs. 34.2%).

**Table 1:** Statistics of factors in patients undergoing baseline biopsies.

Factors	Population	Negative cores	Positive cores (^)
n (%)	945	468 (49,5)	477 (50,7)
**Age, years**			
mean (SD)	66.4 (8.3)	64.4 (8)	68.3 (8.1)
**Body mass index (BMI), kg/m^2^**			
mean (SD)	26.4 (3.2)	26.3 (3.3)	26.5 (3.1)
**Prostate specific antigen (PSA), ng/mL**			
mean (SD)	7.2 (4.5)	6.6 (3.8)	7.8 (5)
**Total Prostate Volume (TPV), mL**			
mean (SD)	42.1 (20.2)	48.3 (22.4)	36 (15.4)
**Central Transition Zone Volume (CTZV), mL**			
mean (SD)	21.1 (13.5)	25.4 (15.6)	16.8 (9.3)
**Peripheral Zone Volume (PZV), mL**			
mean (SD)	21 (8.9)	22.8 (9.4)	19.2 (7.9)
PSA Density (PSAD), (ng/mL)/mL (*)			
mean (SD)	0.19 (0.14)	0.15 (0.09)	0.24 (0.17)
Prostate Volume Index (**)			
mean (SD)	1.04 (0.82)	1.18 (1.08)	0.90 (0.40)
**Digital Rectal Exam (DRE), n (%)**			
normal	637 (67.4)	359 (76.7)	278 (58.3)
abnormal	205 (21.7)	109 (23.3)	199 (41.7)
**Prostatic Chronic Inflammation (PCI), n (%)**			
absent	740 (78.3)	308 (65.8)	432 (90.6)
present	205 (21..7)	160 (34.2)	45 (9.4)
**ISUP grade group**			
1			234 (49.1)
2			110 (23.1)
3			72 (15.1)
4			36 (7.5)
5			25 (5.2)
**Number of Positive Cores (NPC)**			
mean (SD)			4.7 (3.2)

(^) for prostate cancer; (*); ratio of PSA on TPV; (**), ratio of CTZV on PZV; (^), all tests comparing the two groups were significant except for BMI; SD: standard deviation

Analysis of univariate and multivariate linear models are reported in Table-2 . On univariate analysis, all regression coefficients (RC) with relative 95% confidence intervals were significant predictors of the NPC. The regression coefficients were positive for Age (RC 5.8; p <0.0001), PSA (RC 1.3; p <0.0001) and DRE (RC 2.2; p <0.0001), but negative for TPV (RC -2.1; p <0.0001), PVI (RC -1.3; p <0.0001) and PCI (RC -2,1; p <0.0001). On multivariate analysis, two models were computed with model I including Age, PSA, TPV, PVI, DRE and PCI as well as model II considering Age, PSAD, PVI, DRE and PCI. In both models, all factors were independent predictors of the NPC. In model I, regression coefficients resulted positive for Age (RC 4.4; p <0.0001), PSA (RC 1.8; p <0.0001), DRE (RC 1.7; p <0.0001), but negative for TPV (RC -2.1; p <0.0001), PVI (RC -0.6; p=0.002) and PCI (RC -1.4; p <0.0001). Also, in model II, regression coefficients were positive for Age (RC 4.3; p <0.0001), PSAD (RC 1.9; p <0.0001), DRE (RC 1.8; p <0.0001), but negative for PVI (RC -0.7; p <0.0001) and PCI (RC -1.5; p <0.0001). The regression coefficients of PVI and PCI, although decreased when compared to the univariate model, resulted both independent predictors of the NPC.

**Table 2:** Linear regression models of factors predicting the number of positive cores at baseline biopsies in 945 cases.

	Univariate model		Multivariate model (I)		Multivariate model (II)	

Factors	Regression coefficients (95%CI)	P-value	Regression coefficients (95%CI)	P-value	Regression coefficients (95%CI)	P-value
Age (*)	5.8 (4.2 ; 7.4)	<0,0001	4.4 (3.1 ; 5.8)	<0.0001	4.3 (2.9 ; 5.7)	<0.0001
PSA (*)	1.3 (1.0 ; 1.7)	<0.0001	1.8 (1.5 ; 2.1)	<0.0001		
TPV (*)	-2.1 (-2.6 ; -1.7)	<0.0001	-2.1 (-2.6 ; -1.7)	<0.0001		
PSAD (*)	2.1 (1.8 ; 2.3)	<0.0001			1.9 (1.6; 2.2)	<0.0001
PVI (*)	-1.3 (-1.7 ; -0.8)	<0.0001	-0.6 (-1.0 ; -0.2)	0.002	-0.7 (-1.1 ; -0.4)	<0.0001
**DRE**						
normal	Ref		Ref		Ref	
abnormal	2.2 (1.7 ; 2.5)	<0.0001	1.7 (1.4 ; 2.1)	<0.0001	1.8 (1.4 ; 2.2)	<0.0001
**PCI**						
absent	Ref		Ref			
present	-2.1 (-2.5 ; -1.5)	<0.0001	-1.4 (-1.9 ; -1.1)	<0.0001	-1.5 (-1.9 ; -1.1)	<0.0001

See also Table 1; (*) factor evaluated as natural log; CI. confidence intervals

**Figure 2:**
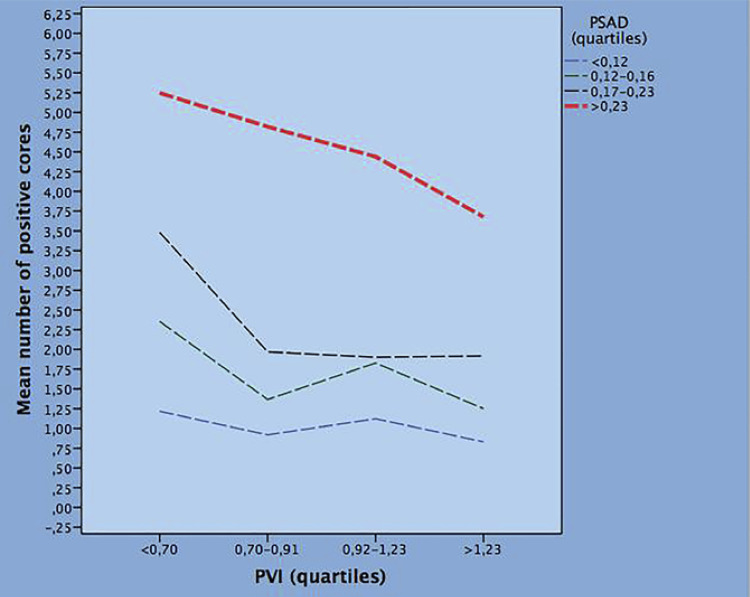
Bivariate model predicting the mean number of positive cores by prostatic specific antigen density (PSAD) and prostate volume index (PVI) quartiles.

**Figure 3:**
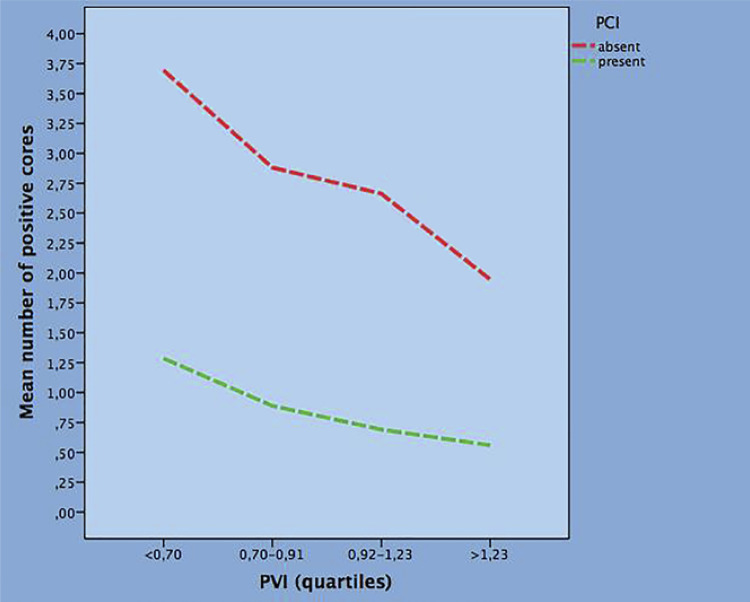
Bivariate model predicting the mean number of positive cores by prostatic chronic inflammation (PCI) and prostate volume index (PVI) quartiles.

Table-3 shows bivariate clinical models of factors predicting the mean NPC. In each model, PVI, evaluated as a continuous variable, is combined with a clinical factor which is stratified into quartiles with the first quartile as reference. In each model, PVI decreases the mean rates of NPC. Considering positive predictive factors along groups, the mean NPC was increased by Age, PSAD, abnormal DRE and PSA, but only for values above the third quartile (PSA >8.4ng/mL). Evaluating negative predictors along quartiles, the mean NPC was decreased by TPV and PCI. The association of mean NPC with PSAD (positive) and PVI quartiles (negative) is depicted in Figure 2 . Finally, Figure-3 shows that the presence or absence of PCI decreases or increases the mean NPC along PVI quartiles.

## DISCUSSION

BPH and PCA are age related disease which may be both present when evaluating patients ( 1 , 2 ). Abnormal clinical findings trigger baseline biopsies because PCA is suspected. When PCA is ruled out, tumor grade and intra-prostatic tumor load are pivotal parameters for classifying patients into risk categories which impact on management decisions. In the low and intermediate risk categories, tumor burden, which is evaluated as stage T1c or T2 (a/b), is a critical issue because cancer biology is not properly assessed as documented by high upstaging and upgrading rates after radical prostatectomy. Age, abnormal DRE, PSA, TPV and PSAD are known factors that associate with PCA risk at baseline biopsies, moreover, each factor relates to tumor load ( 1 ).

**Figure 4:**
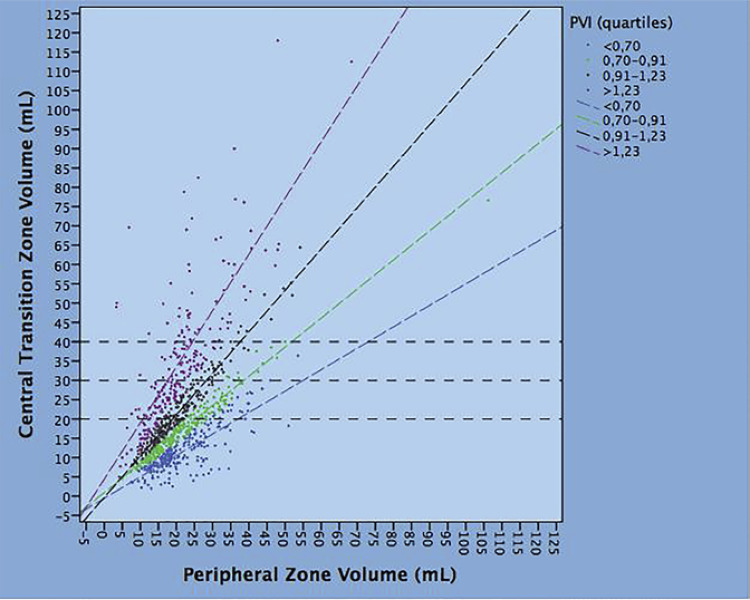
The regression lines of the transitional zone volume (TZV) as a function of the peripheral zone volume (PZV). The patients are classified into 4 groups according to PVI quartiles. The different relations between volumes are outlined along different PVI quartiles.

The prostate volume has been demonstrated to have an inverse correlation with prostate cancer risk ( 15 - 20 ). In our study, we focused on evaluating all these factors together with PVI in order to evaluate tumor biology which was assessed as tumor load by the NPC. NPC was independently decreased by PVI indicating inverse association between PVI and tumor load. This finding was expected since we have previously shown that PVI associated with a decreased risk of PCA at baseline biopsies ( 7 , 13 , 14 ). So far, PVI associated with a decreased risk of PCA and decreased NPC in patients undergoing baseline biopsies indicating inverse association with tumor biology. PVI is a pure measure since represents a ratio between volumes. We have shown that PVI represents the gradient of the regression line of TZV as a function of PZV ( 7 , 13 , 14 ). Considering the relations between PZV and TZV, PVI quartiles represent the different gradients of the regression lines between the two volumes. This is illustrated in Figure-4 which shows the regression lines of TZV as a function of PZV. As shown, the patients are classified into 4 groups according to PVI quartiles. The different relations between volumes are outlined along different PVI quartiles. As an example, when TZV is measured 40mL, the mean PZV is 20mL for PVI >1.23, 38 for PVI between 0.91-1.23, 5, 52 for PVI between 0.70-0.91 and 75 for PVI <0.70; so far, when TZV is fixed constant, PZV increases along decreasing PVI quartiles. This indicates that, for fixed values of TZV, the mean rates of NPC are increasing for increasing values of PZV which decreases PVI, as shown by the results of the study. Our findings suggest that TPV is not to be considered just a measure but the sum of a combination of non-homogenous volumes including the two main zones of the prostate.

The dynamics of the two zonal volumes, CTZV and PZV, change with time indicating a close association with aging and PCA risk. These findings suggest a new way to approach the subject of dealing with the biology of tumors of the prostate gland. The inverse association of PVI with PCA biology may be explained by theories suggesting associations between growth and differentiation of the prostate. During the aging process, CTZV and PZV of the prostate are exposed to different levels of androgenic activity such as total testosterone which determine different dynamics on volume growth rates. In theory, higher testosterone activity in the PZV might trigger larger growth rates than CTZV leading to decreased PVI values. Moreover, since the peripheral zone is being exposed to higher testosterone levels, tumors with more aggressive biology are expected to occur in this zone. This hypothesis is supported by findings showing positive association between preoperative total testosterone levels more aggressive tumors in radical prostatectomy specimens ( 21 - 23 ).

Also, there may be an increased chance of accurately targeting a cancer lesion in patients with smaller prostates when compared to patients with larger prostates with similarly sized lesions. This may also be the reason why lower cancer detection rates are reported in patients with large prostates. However, this theory is in contention and has not been proven in the literature ( 24 ), therefore in our clinical practice we did not increase the number of biopsy cores according to the prostate volume.

Importantly, although there has been a recent increase in the utilization of prostatic MRI in the last few years, ( 25 ) TRUS is a more cost-effective and widely-available imaging modality that can be used to evaluate the prostate volume in primary, secondary and tertiary centers. On the other hand, TRUS volume evaluation using the ellipsoid formula has been related to 15% intra-observer variability and 93% reliability, as well as 22% of inter-observer variability and 87% inter-observer reliability ( 26 ).

When planning baseline biopsies because of suspected cancer, high rates of negative cases are to be expected, moreover, negative cancer outcomes arise the unsettled issue of how to avoid unnecessary biopsies ( 1 , 2 ). It has been shown that large prostates are an increased risk of unnecessary biopsies because they associate with higher PSA values at diagnosis ( 19 ). Moreover, prostatic chronic inflammation type IV is also a feature of unnecessary baseline biopsies because the condition associates with both increased PSA levels and/or abnormal DRE ( 4 ). Literature reviews on this subject have shown that the risk of PCA is reduced when PCI is present in prostate microenvironment ( 5 , 27 ). Our group has shown that PCI associates with a reduced risk of PCA at baseline biopsies ( 6 - 12 ). In the present study, we wanted to test the hypothesis that the presence of PCI in prostate microenvironment could associate with less aggressive tumor biology. The results showed that the mean rate of NPC was decreased when PCI, which represented 21% of the population, was detected in biopsy cores. This result was expected after we have shown the inverse association between PCI and tumor biology defined by ISUP grade groups ( 12 ). An unexpected and surprising finding was that both PVI and PCI independently decreased the mean rate of NPC. So far, PCI inversely related to PCA biology because it associated with less extensive tumor load independently by PVI measurements. These findings are depicted in Figure-3 which shows the phenomena involving PVI and PCI in PCA biology. It is interesting to speculate on hypotheses explaining the negative association between PCI and PCA. As a theory, PCI might be actively involved in the early steps of PCA by inducing the differentiation of anti-tumorigenic cellular phenotypes by the immune system in prostate microenvironment ( 28 , 29 ). We have hypothesized cellular signalling pathways between PCI and PCA ( 6 - 12 ). Briefly, during the first steps of carcinogenesis, high grade PIN interrupts the basement membrane with diffusion of cancer cells that induce recruitment of immune cells by producing inflammatory factors and cytokines. Going on with this patterns, tumor antigens are exposed to lymphocytes which include both the helper (CD4+) and cytotoxic (CD8+) phenotypes which cooperate to each other in order to kill the early transformed cancer cells. The result is that cancer progression is impaired or slowed down by the activated immune system.

We have also shown that PCI is related to prostate volumes as well as PCA ( 11 ). The association of PCI was positive with CTZV and negative with PCA. In the present study we have shown that increasing PVI measurements decreased the NPC indicating inverse association with aggressive tumor biology. We may speculate interactions between PCI and CTZV growth rates which are mediated by biological factors produced by inflammatory cells, total testosterone and estradiol intra-prostatic levels. Interactions and variations among these factors may induce and accelerate CTZV growth rates which prevail on those ongoing in the PZV which is compressed by the expanding CTZV. Rapid and increasing growth rates involving CTZV increase PSA production which leads the clinician to plan baseline biopsies which are less likely to be positive or to have an aggressive biology in prostates harboring these features. Controlled studies are required in order to verify these hypotheses on biology of prostate volumes, PCI and PCA.

In clinical practice, total PSA is an important parameter for assessing prostate diseases because it relates to prostate volumes, cancer and chronic inflammation; moreover, PSAD has a limited power in predictive PCA because it is closely related to prostate volumes ( 1 , 2 , 19 ). So far, increased PSA values may be sustained by one or more of these conditions. In our study, we have shown that although continuous PSA was an independent predictor of NPC, only values above the third quartile (PSA >8.4ng/mL) significantly associated with tumour extension ( Tables 2 and 3 ), interestingly, the mean rate of positive cores decreased from 6 to 2 when PVI increased from the first to the third quartile in this set of patients. PSA measurements below the fourth quartile did not predict NPC because these values might be related to prostate volumes and/or chronic inflammation, moreover, it is possible that the fraction of PSA related to tumor load was so low that it did allow significant predictive value. On the contrary, we suppose that PSA values >8.4 were predictive because they associated with higher tumor burden as shown by the mean NPC.

**Table 3:** Bivariate linear regression models of factors predicting the number of positive cores.

Factors	Regression coefficients (95% CI)	P-value
PVI (*)	-1.4 (-1.8 ; -1.1)	<0.0001
Age by quartiles (**)		
<62	Ref	
62-67	0.7 (0.2 ; 1.3)	0.007
68-72	1.1 (0.5 ; 1.7)	<0.0001
>72	2.1 (1.5 ; 2.7)	<0.0001
PVI (*)	-1.4 (-1.9 ; -1.1)	<0.0001
**PSA by quartiles**		
<4.8	Ref	
4.8 - 6.2	0.2 (-0.2 ; 0.8)	0.332
6.3 - 8.4	0.3 (-0.2 ; 0.9)	0.223
>8.4	2.0 (1.4 ; 2.6)	<0.0001
PVI (*)	-0.7 (-1.1 ; -0.2)	<0.0001
**TPV by quartiles**		
<28.2	Ref	
28.2 - 37.9	-0.7 (-1.2 ; -0.1)	0.016
38 - 51.5	-1.3 (-1.9 ; -0.7)	>0.0001
>51.5	-2.1 (-2.6 ; -1.4)	<0.0001
PVI (*)	-0.7 (-1.2 ; -0.3)	<0.0001
**PSAD by quartiles**		
<0.12	Ref	
0.12 - 0.16	0.6 (0.1 ; 1.1)	0.023
0.17 - 0.23	1.2 (0.7 ; 1.7)	<0.0001
>0.23	3.5 (2.9 ; 4.1)	<0.0001
PVI (*)	-1.2 (-1.5 ; -0.7)	<0.0001
**DRE**		
normal	Ref	
abnormal	2.0 (1.6 ; 2.4)	<0.0001
PVI (*)	-1.1 (-1.4 ; -0.6)	<0.0001
**PCI**		
absent	Ref	
present	-1.8 (-2.3 ; -1.4)	<0.0001

See also Table 1; (*) evaluated by natural logs

All these findings are more important considering that in our previous experience we demonstrated that the NPC is strongly associated with more aggressive PCA resulting in tumor upgrading and upstaging, unilateral or bilateral lymph node metastasis and seminal vesical invasion ( 30 - 34 ).

These results should be considered in clinical practice in order to avoid unnecessary baseline biopsies.

Our study has many strengths. First, it represent the results of a single center in which cores were evaluated by a single dedicated pathologist. Second, all biopsies were baseline and taken in a standard fashion with the standard number of 14 cores which were random and representing different coded zones of the prostate. Third, the analysis did not consider targeted cores in order to avoid skewing phenomena. Fourth, TPV and CTZ volumes were measured in standard fashion in each patient by trained urologists in performing trans-perineal prostate biopsies. Fifth, PCI was investigated in each core in a standardized fashion as reported in the methods section. Sixth, the parameters assessed are useful for evaluating tumor extension by the NPC.

However, our study also has several limitations. First, because it was retrospective and not prospective, it has all the limits related to such kind of studies. Second, prostate volume evaluations were performed using ellipsoid -TRUS method that has been demonstrated to have a non-negligible intra and inter observer variability ( 26 ). Third, because prostate volumes were not compared to prostate weights in radical prostatectomy specimens, measured prostate volumes might not reflect the true values of prostate sizes. Fourth, tumor extension was not compared to PCA volume in radical prostatectomy specimens. Fifth, larger prostates with higher PSA levels might undergo biopsy more frequently than smaller prostates with lower PSA levels and this might be a bias. Fifth, PCI was not graded and inflammatory cells were not qualitatively assessed for immunologic components. Finally, comparative studies are missing.

## CONCLUSIONS

In patients undergoing baseline prostate biopsies, PVI and PCI decreased the number of positive cores and associated with less aggressive tumor biology expressed by lower tumor extension inside the gland. PVI is a parameter to be considered before planning baseline random biopsies. Confirmatory studies are required.
